# ^1^H NMR-Based Profiling Reveals Differential Immune-Metabolic Networks during Influenza Virus Infection in Obese Mice

**DOI:** 10.1371/journal.pone.0097238

**Published:** 2014-05-20

**Authors:** J. Justin Milner, Jue Wang, Patricia A. Sheridan, Tim Ebbels, Melinda A. Beck, Jasmina Saric

**Affiliations:** 1 Gillings School of Global Public Health, Department of Nutrition, University of North Carolina at Chapel Hill, Chapel Hill, North Carolina, United States of America; 2 Section of Computational and Systems Medicine, Department of Surgery and Cancer, Faculty of Medicine, Imperial College, London, United Kingdom; University of North Carolina at Greensboro, United States of America

## Abstract

Obese individuals are at greater risk for death from influenza virus infection. Paralleling human evidence, obese mice are also more susceptible to influenza infection mortality. However, the underlying mechanisms driving greater influenza severity in the obese remain unclear. Metabolic profiling has been utilized in infectious disease models to enhance prognostic or diagnostic methods, and to gain insight into disease pathogenesis by providing a more global picture of dynamic infection responses. Herein, metabolic profiling was used to develop a deeper understanding of the complex processes contributing to impaired influenza protection in obese mice and to facilitate generation of new explanatory hypotheses. Diet-induced obese and lean mice were infected with influenza A/Puerto Rico/8/34. ^1^H nuclear magnetic resonance-based metabolic profiling of urine, feces, lung, liver, mesenteric white adipose tissue, bronchoalveolar lavage fluid and serum revealed distinct metabolic signatures in infected obese mice, including perturbations in nucleotide, vitamin, ketone body, amino acid, carbohydrate, choline and lipid metabolic pathways. Further, metabolic data was integrated with immune analyses to obtain a more comprehensive understanding of potential immune-metabolic interactions. Of interest, uncovered metabolic signatures in urine and feces allowed for discrimination of infection status in both lean and obese mice at an early influenza time point, which holds prognostic and diagnostic implications for this methodology. These results confirm that obesity causes distinct metabolic perturbations during influenza infection and provide a basis for generation of new hypotheses and use of this methodology in detection of putative biomarkers and metabolic patterns to predict influenza infection outcome.

## Introduction

Obesity has reached epidemic proportions [1]. Global approximations report that two out of every three individuals are clinically overweight (BMI≥25–29.9) or obese (BMI≥30) [2]. The pathological complications of obesity are diverse and include co-morbidities such as cardiovascular disease, type 2 diabetes, and hypertension, to name a few [3], [4]. Obese individuals are also more susceptible to viral and bacterial infections [5]. In 2009, the Centers for Disease Control and Prevention reported a high prevalence of obesity among intensive care patients with confirmed 2009 pandemic H1N1 influenza (pH1N1) infection [6]. Several investigations have since established obesity as an independent risk factor for enhanced pH1N1 [6]–[10] and seasonal influenza infection severity [11]–[13]. Given that seasonal influenza epidemics result in 250,000 to 500,000 deaths globally [14] and future pandemics are likely imminent [15], understanding how obesity enhances influenza severity is a global public health concern.

We have previously shown that obese individuals exhibit impaired cellular and humoral immune responses to influenza vaccination [16]. Further, mouse models of obesity have extensively demonstrated that obesity enhances influenza infection mortality [17]–[23]. Both innate and adaptive influenza immune defenses are altered by obesity, but elucidation of the underlying mechanisms driving greater influenza severity in obese mice is currently lacking [5], [19]–[21], [24]. Further, obesity is inherently a metabolic disease, and thus far, no studies have considered how dynamic changes in metabolism during influenza may impact immunity and infection outcome in the obese.

Metabolic profiling, combining ^1^H nuclear magnetic resonance (NMR) spectroscopy and multivariate statistical data analysis, has found wide application in metabolic syndrome related diseases and has also gained significant momentum in infectious diseases for discovery of predictive and diagnostic biomarkers [25]–[29]. Although few studies have utilized metabolic profiling to investigate influenza pathogenesis, it has been reported that infection with the mouse adapted strain, influenza A/FM/1/47, caused distinct perturbations in fatty acid and amino acid metabolism in the serum of infected mice compared with uninfected mice [30]. In more recent years, the approach has evolved towards a more systemic assessment of host responses, and integrating immune measures has become established in particular [31], [32]. The purpose of this study was to develop a global view of the impact of obesity on metabolism and immunity during influenza infection and to generate data which can serve as the basis for the formation of new mechanistic hypotheses and perhaps new prognostic or diagnostic methodology.

In the present study, lean and obese mice were infected with a mild dose of influenza A/PR/8/34 (H1N1) and subsequently tissues and biofluids were harvested for metabolic profiling.^ 1^H NMR-based profiling revealed distinct metabolic signatures between infected lean and obese mice. Interestingly, we identified unique metabolic signatures in urine and fecal samples that allowed for discrimination of infection status and distinguished uninfected and infected lean and obese mice. Further, the metabolic analysis was extended to include lung tissue, bronchoalveolar lavage fluid (BALF), serum, mesenteric white adipose tissue (WAT), and liver from infected lean and obese mice. We detected significant alterations in ketone body, lipid, choline, nucleotide, vitamin, amino acid and carbohydrate metabolic pathways in influenza infected obese mice. We also analyzed T cell populations from lean and obese infected mice to identify immune-metabolic correlation structures, and several interactions between a variety of metabolites and BAL or draining lymph node T cell populations were uncovered. Identification of differential metabolic signatures and immune-metabolic structures in lean and obese mice facilitates the establishment of metabolic profiling as a useful tool for characterizing influenza infection responses and advances the current understanding of potential factors contributing to impaired influenza protection in obese mice.

## Methods

### Ethics Statement

Animal experiments were conducted at the University of North Carolina at Chapel Hill Animal Facility, which is fully accredited by the American Association for Accreditation of Laboratory Animal Care. All mouse-related procedures were approved by the University of North Carolina at Chapel Hill Institutional Animal Care and Use Committee.

### Animal Housing and Maintenance

Weanling, male, C57BL/6J mice (Jackson Laboratory, Bar Harbor, ME) were housed under pathogen free conditions and fed a high fat (45% kcal fat, Research Diets D12451) or low fat, control diet (10% kcal fat, Research Diets D12450B, New Brunswick, NJ) for 20 weeks.

### Reagents

Phosphate buffer for ^1^H NMR acquisition of urine and extracts was made at a D_2_O∶H_2_O ratio of 1∶1 at a pH of 7.4 containing Na_2_HPO_4_ and NaH_2_PO_4_ (Sigma-Aldrich). D_2_O served as a field frequency lock and sodium 3- (trimethylsilyl) [2,2,3,3-2H4] propionate (Sigma-Aldrich) at 0.01% was added as a chemical shift reference represented at δ 0.0. For ^1^H NMR acquisition of serum 0.9% saline was equally made up in 50% D_2_O (Sigma-Aldrich). HPLC-grade water, chloroform, and methanol, for tissue extractions were obtained from VWR International Ltd.

### Influenza Infection and Sample Collection

Influenza A/Puerto Rico/8/1934 (H1N1, PR/8), (ATCC, Manassas, VA), was propagated in 10–12 day old embryonated chicken eggs as described previously [19], [21]. At 72 h post-infection, allantoic fluid was harvested, clarified by centrifugation and then stored at −80°C [21]. For influenza inoculations, mice were lightly anesthetized via isoflurane inhalation, and infected with 1.1×10^2^ 50% tissue culture infective dose (TCID_50_) PR/8 in 50 µL of phosphate buffered saline. The mice were infected via non-invasive oropharyngeal aspiration, as similarly reported [21], [33], [34]. Mice were sacrificed by cervical dislocation.

Urine and fecal samples for metabolic profiling were obtained at three different time points: one day prior to infection (D-1), 2 and 6 days post-infection (dpi). Details regarding fecal/urine sample collection are described elsewhere [27]. At 9 dpi, liver, lungs, mesenteric (visceral) white adipose tissue (WAT), bronchoalveolar lavage fluid (BALF) and mediastinal lymph nodes (mLN) were harvested and further processed for flow cytometry or were flash frozen in liquid nitrogen. Collection of BALF was performed as previously described [19], [21]. Urine, serum, fecal pellets, BALF, liver, lung and WAT samples were shipped to Imperial College London on dry ice for ^1^H NMR data acquisition and analysis.

### Flow Cytometry

BAL and mLN cells were processed and stained for flow cytometry as previously described [19], [21] with the following antibodies: CD16/32 (Fc blocker), CD4 (FITC), CD25 (PE-Cy7), and Foxp3 (APC) from eBioscience (San Diego, CA); CD3 (APC/Cy7) and CD8 (PerCP) from BioLegend (San Diego, CA). The MHC class I tetramer (PE) specific for H-2Db-resricted epitope of the influenza nucleoprotein (NP, D_b_NP_366-74_) of PR/8 was obtained from the NIH Tetramer Core Facility (Atlanta, GA). Stained cells were subsequently analyzed on a CyAn ADP Analyzer flow cytometer (Beckman Coulter, Inc., Fullerton, CA), and all flow cytometry data were analyzed via FlowJo Software (TreeStar, Ashland, OR). All flow cytometry analysis of T cells consisted of a doublet exclusion gate followed by gating of CD3^+^ cells for further analysis of CD4^+^ and CD8^+^ T cell populations.

### Sample Preparation and Acquisition of ^1^H NMR Spectral Data

Urine, feces, serum, and tissue samples were prepared according to previously published protocols [27], [35]. Individual BALF samples (200 µl) were mixed with phosphate buffer (400 µl) and 550 µl of the mixture was subsequently transferred into 5 mm diameter standard NMR tubes for data acquisition (Bruker Biospin, Rheinstetten). All biological samples were analyzed using a 600 MHz Avance DRX NMR spectrometer (Bruker; Rheinstetten), employing a standard one dimensional (1D) ^1^H NMR Noesypr1d pulse sequence with water suppression (recycle delay (rd)-90°-t1-90°-tm-90°-acquisition time) for all samples. Recycle delay (rd) and mixing time (tm) were set to 2 s and 100 ms respectively. For obtaining more comprehensive information on the serum samples, two additional pulse programs were utilized namely Carr-Purcell-Meiboom-Gill (cpmgpr) and diffusion edited spectroscopy (ledbipolpr) [35]. The numbers of scans were adjusted for each biological matrix, whereby urine and serum were analyzed in 256 and extracts in 128 scans to obtain maximum signal output. Data from each sample was accumulated into 32 k data points within a spectral width of 20 ppm.

### Pre-processing of Spectral Data

Topspin 3.1 (Bruker Biospin) was utilized for partially pre-processing of the raw spectra, including manual phasing, baseline correction and calibration of the chemical shifts to the TSP signal at δ 0.00 or to the lactate doublet at 1.33 representing the CH_3_ signal, for serum samples. Spectra were subsequently imported into a MATLAB interface (Mathworks Inc., USA) where regions containing ethanol, methanol, urea, and water resonances were cut out from the whole spectra. Selected regions were δ 1.049–1.232, δ 3.599–3.7 (ethanol) for BALF spectra, δ 4.704–6.318 (H_2_O and urea) for urine, δ 3.332–3.443 (methanol), δ 4.598–5.001 (H_2_O) for serum, δ 4.216–5.556 (H_2_O) for feces, δ 4.723–5.012 (H_2_O) for liver, δ 4.713–5.075 (H_2_O) for lung, and δ 4.675–5.093 (H_2_O) for WAT. All remaining spectral regions were aligned and normalized to the median spectrum according to an in-house developed MATLAB script (Dr. Kirill Veselkov) [36].

### Multi- and Uni-variate Data Treatment

Processed spectral data were exported to SIMCA-P software (Umetrics, Sweden) for Principal component analysis (PCA) and projection to latent structure discriminant analysis (PLS-DA) for obtaining an overview on the data distribution and presence of class differences [37], [38]. For identification of the discriminatory metabolites, orthogonal PLS-DA (O-PLS-DA) was applied using an in-house developed script in MATLAB including a 7-fold cross validation [39]. Metabolites that are related to a certain class are represented by yellow to red colors in the color code included in the O-PLS-DA plots, whereby the exact correlation value can be extracted from the main plot in MATLAB. Here, only candidate biomarkers above a correlation threshold corresponding to p≤0.05 were selected as significant.

In order to explore correlation structures between metabolic data and T cell populations, an additional correlation analysis was conducted. The spectral information which built the X-matrix was therefore integrated with a second Y-matrix that contained the flow cytometry data. The Pearson-based correlation script further contained a peak picking algorithm to reduce inclusion of noise, and a 10,000 fold permutation resulting in display of correlations with p≤0.05 only. Identity of peaks was confirmed by using in-house NMR chemical shift database, Chenomx NMR suite profiler software (Chenomx Inc, USA), and published ^1^H NMR assignments [27], [40], [41]. Identities were further confirmed by using statistical total correlation spectroscopy (STOCSY) in a MATLAB environment [39].

## Results

### Metabolic Profiling Uncovers Distinct Metabolic Perturbations in Influenza Infected Obese Mice

**Figure 1:**
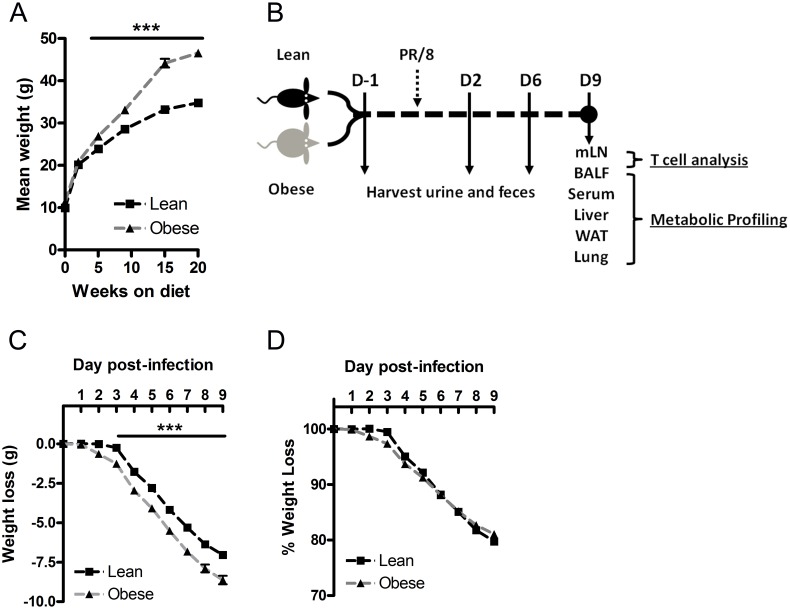
Summary of influenza infection and metabolic profiling model. A) Weanling, male C57BL6/J mice were maintained on a high fat (45% kcal fat) or low fat control diet (10% kcal fat) for 20 wks, n≥9. B) Timeline of samples harvested for metabolic profiling. Lean and obese mice were infected with 1.1×10^2^ TCID_50_ of influenza A/PR/8/34, and urine and feces were collected at -1 (uninfected mice), 2 and 6 dpi. Terminal samples (mLN, BALF, serum, liver, WAT and lungs) were collected from the same cohort of mice at 9 dpi. Flow cytometry was used to enumerate BAL and mLN T cell populations for immune-metabolic integration, n = 8–9. C) Absolute weight loss in lean and obese mice following PR/8 infection, n≥9. D) Percent weight loss normalized to pre-infection body weight, n≥9. Values represent mean 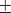
SEM, ***p<0.0005 compared with lean mice.

Weanling, male C57BL/6J mice were maintained on a high fat (45% kcal fat) or a low fat, control diet (10% kcal fat) for 20 weeks. Following the dietary exposure, obese mice weighed 33% more than lean mice (Figure 1A). Figure 1B is a representative timeline for collection of samples processed for immune-metabolic characterization, and all samples were obtained from the same cohort of lean and obese mice. Following the 20 wk dietary exposure, mice were infected with 1.1×10^2^ TCID_50_ of PR/8. Although obese mice lost more absolute weight compared with lean mice (Figure 1C), there were no differences detected in percent weight loss between the two groups (Figure 1D). It is well established that obese mice are more likely to die from influenza if given a sufficient viral dose [17]–[20], [23]. However, we utilized a relatively mild infection dose, as only 10% of lean and obese mice succumbed to the infection. We chose a mild infection for this model in order ensure that we captured metabolic changes induced by obesity during infection rather than secondary responses caused by lethal infection conditions.

**Figure 2:**
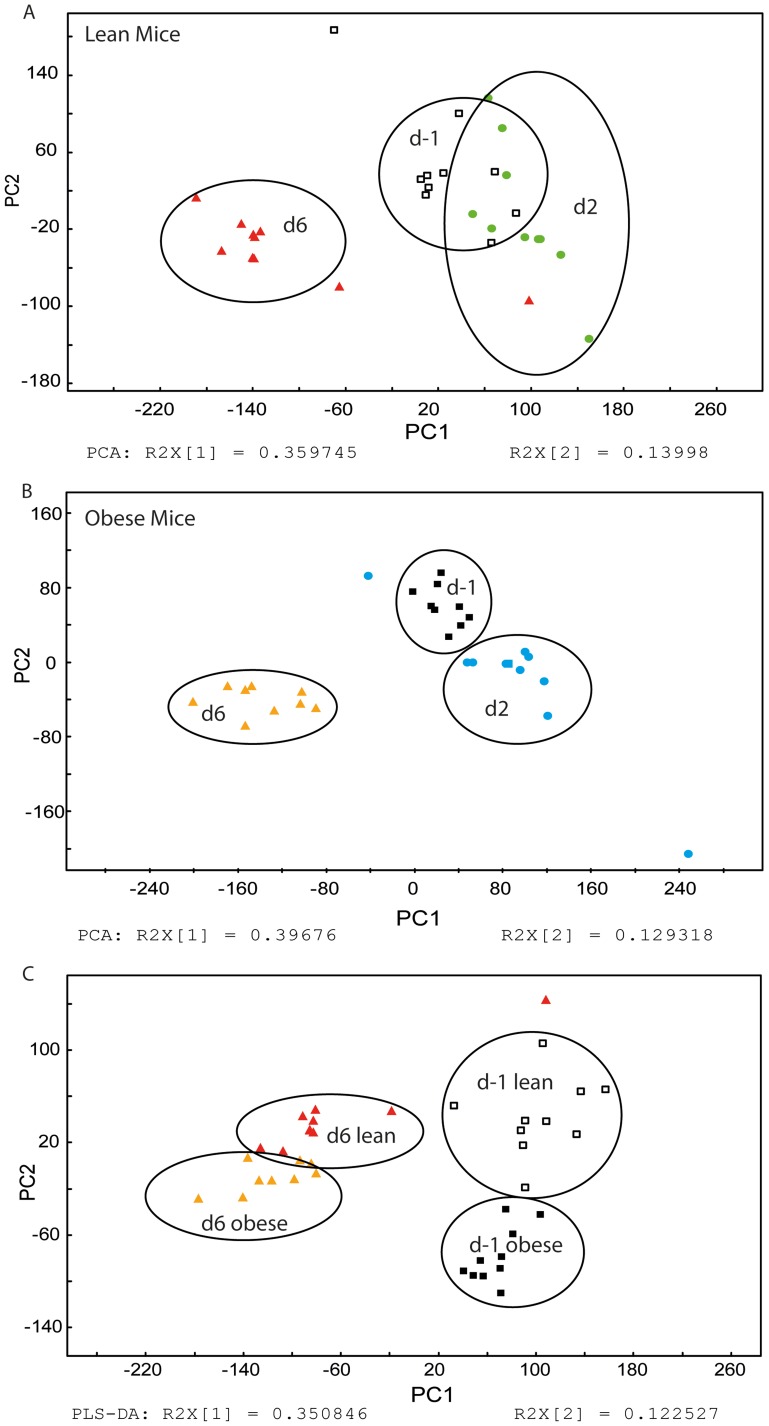
Pre-processed ^1^H NMR urine spectra were analyzed using PCA and PLS-DA. Urine spectra from lean and obese mice were collected one day prior to infection and at 2 and 6 dpi. A/B) The urine spectra from lean mice (A) and obese mice (B) showed clear separation according to time, in a PCA analysis. C). PLS-DA analysis showed additional separation between lean and obese mice for two selected time points (i.e. -1 dpi and 6 dpi). n = 8–9.

We first set out to determine if metabolic profiling could distinguish urine and fecal samples from uninfected and infected lean and obese mice. We chose urine and feces because these are biosamples that could easily be tested in humans. Further, we chose to sample urine and feces at 2 dpi and 6 dpi to capture metabolic changes during early and late infection responses. Figure 2 represents a PCA scores plot showing clear separation of naïve (D-1) urine samples from early infection (D2) and later infection (D6) time points for lean (Figure 2A) and obese mice (Figure 2B). Pre-infection and 6 dpi samples were furthermore combined in a PLS-DA analysis in order to capture whether obesity status or infection state was the predominant influence to the murine metabolic profile (Figure 2C). Of interest, all infection-dietary groups displayed metabolically distinct urinary profiles.

Analysis of urinary and fecal metabolites differentially altered in lean and obese mice during the infection revealed mixed effects in a variety of metabolic processes, including perturbations in nucleotide, vitamin, ketone body, carbohydrate, choline and lipid metabolism (Table 1). Reflective of impaired glucose tolerance, characteristic of obesity, obese mice had greater urinary glucose levels prior to infection and throughout the course of the infection [42], [43]. Further, uninfected obese mice had significantly higher concentrations of urinary taurine, ureidopropanoate, 1-methylnicotinamide, several unknown metabolites and lower levels of trimethylamine. At 2 dpi ascorbate, ureidopropanoate and acetylcarnitine were detected in the urine of obese mice, whereas trimethylamine levels were greater in lean mice at -1 and 2 dpi. Urinary 3-hydroxybutyrate concentration was greater at 6 dpi in obese mice as well as several lipid metabolites in fecal extracts at 2 dpi.

**Table 1:** Metabolic biomarkers recovered from urine and fecal extracts during influenza infection in lean and obese mice^a^.

Metabolic Pathway	Metabolite	Day -1	Day 2	Day 6
**Ketone Body Metabolism**	3 hydroxybutyrate			**Urine**
**Lipid Metabolism**	acetylcarnitine		**Urine**	
	(CH2)n		**Feces**	
	**CH2**CH2CO		**Feces**	
	CH2**CH2**CO		**Feces**	
	propionate		**Feces**	
	taurine	**Urine**		
**Choline Metabolism**	choline	**Feces**		
**Microbial Metabolism**	trimethylamine	Urine	Urine	
**Nucleotide Metabolism**	ureidopropanoate	**Urine**	**Urine**	
**Vitamin Metabolism**	ascorbate		**Urine**	
	1-methylnicotinamide	**Urine**		
**Carbohydrate Metabolism**	glucose	**Urine**	**Urine**	**Urine**
**UK1**	2.458(s)		**Feces**	
**UK2**	8.54(d), 8.33(d), 6.7(d), 6.67(d), 3.65(s)	**Urine**	**Urine**	**Urine**

a Bolded and underlined urine and feces indicates that metabolite was significantly higher in obese mice, and metabolites in normal font were significantly lower in obese mice. UK: unknown metabolite. n = 8–9.

**Figure 3:**
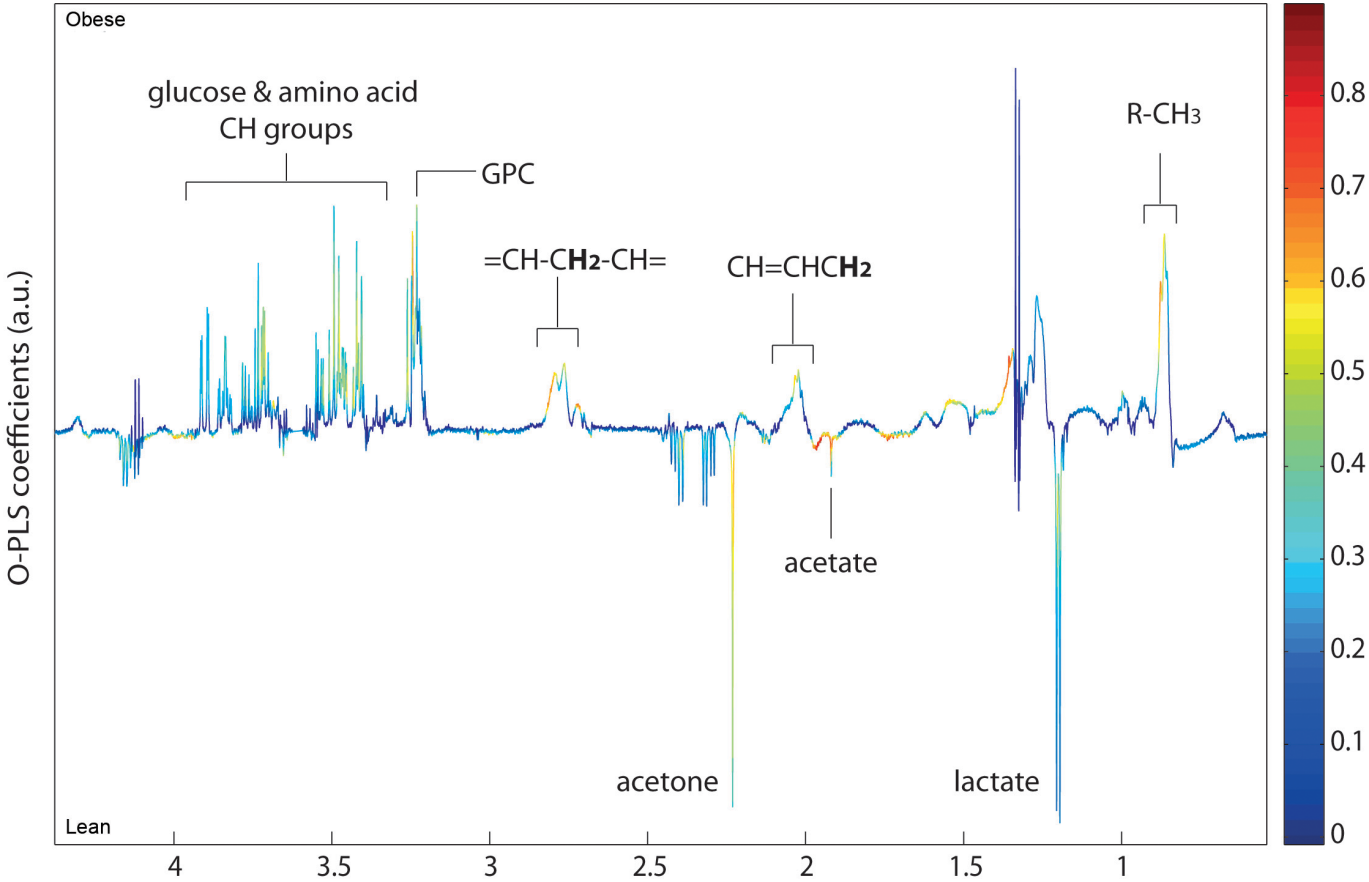
O-PLS-DA analysis comparing serum ^1^H NMR spectra of lean and obese mice at 9 dpi. n = 8–9.

The metabolic analysis was also extended to include assessments of BALF, lung tissue, serum, liver and mesenteric WAT from lean and obese mice at 9 dpi (Table 2). To limit variation within dietary groups, due to intra-individual differences between mice, all samples used for profiling were obtained from the same cohort of mice. Although this is an inherent strength in our model, it limited the analysis to one time period for harvesting terminal samples (serum, WAT, BALF, lung and livers). We specifically chose BALF and lung to examine local changes at the site of infection, and we included peripheral tissues and serum to enhance understanding of the systemic and global consequences of obesity during influenza. Analyses of tissues at 9 dpi revealed that WAT showed the greatest number of altered metabolites, whereby the majority of changes were linked to amino acid metabolism (Table 2). Additionally, serum showed a high degree of metabolic change between lean and obese mice, including relatively higher general lipid levels in the obese mice (Figure 3, Table 2). However, lean mice had greater levels of serum acetone, 3-hydroxybutryate and acetate. Lung and BALF did not show any significant differences between lean and obese mice at 9 dpi.

**Table 2:** Discriminatory metabolites between lean and obese mice at 9 days post-infection in liver, serum and white adipose tissue samples^a^.

Metabolic Pathway	Metabolite	Liver	Serum	WAT
**Amino acid Metabolism**	alanine			**+**
	glutamate			**+**
	isoleucine			**+**
	leucine			**+**
	lysine			**+**
	methionine			**+**
	phenylacetylglycine			**+**
	tyrosine			**+**
	valine			**+**
**Biosynthesis of Secondary Metabolism**	*scyllo*-inositol			**−**
**Carbohydrate Metabolism**	glucose	**+**	**+**	
**Ketone Body Metabolism**	acetone		**−**	
	3 hydroxybutyrate		**−**	
**Lipid Metabolism**	glycerophosphocholine		**+**	
	LDL ( = CH-CH2-)		**+**	
	LDL (-CH = CH-CH2-CH = CH-)		**+**	
	LDL (CH3)		**+**	
**Pyruvate Metabolism**	acetate		**−**	

a Metabolites that were detected at a significantly greater level in obese mice are indicated by +, whereas − represents metabolites detected at lower levels in obese mice compared with lean mice, n = 8–9.

### Differential Immune-metabolic Correlations in Influenza Infected Lean and Obese Mice

Metabolic profiling has previously been used to explore immune regulatory systems during infections by applying multivariate statistical methods to uncover metabolic-immune interactions [32],[44]. To further assess the co-variation between tissue/biofluid metabolites and immune parameters in our model, a correlation analysis was conducted between T cell population numbers and ^1^H NMR data for lean and obese statuses separately in order to characterize the systemic background metabolism linked to T cell responses. Although a variety of cellular defenses are altered by obesity, we chose to focus our analysis on T cell populations because this cell type is consistently altered by obesity during influenza infection [17], [19], [21], [24], [45]. We have previously detected perturbations in antigen specific CD8^+^ T cell and regulatory T cell (Tregs) responses during influenza infection in obese mice [19]–[21], [45]; therefore, we focused our immune-metabolic integration on these cell types in particular. For influenza-specific CD8^+^ T cells, we utilized an MHC class I tetramer specific for H-2Db-resricted epitope of the influenza nucleoprotein (NP, D_b_NP_366-74_) of PR/8, and CD4^+^ Tregs were identified by expression of the transcription factor Foxp3.

**Figure 4:**
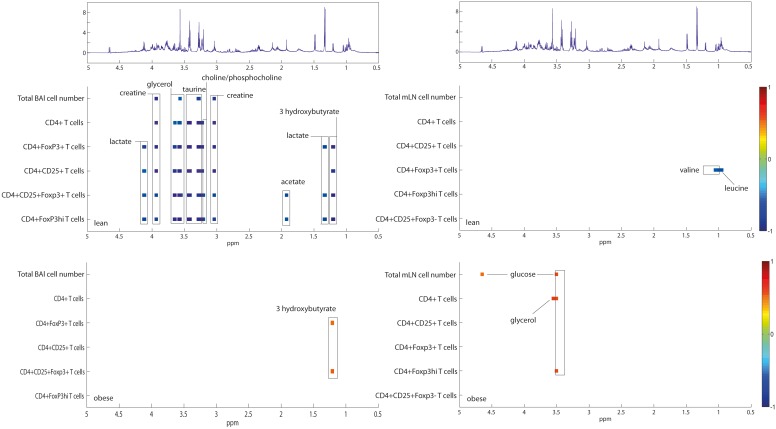
Correlation analysis of lung ^1^H NMR data and T cell populations in BALF (left panel) and mLN compartments (right panel) reveals differential correlation patterns between lean and obese mice. n = 8–9.

The ^1^H NMR data from each biological compartment (i.e. lung, liver, WAT, feces, BALF, serum, and urine) were correlated with BAL and mLN T cell populations at 9 dpi. Urine (6 dpi), lung (Tables 3 and 4) and liver presented the highest amount of T cell-metabolite correlations in both lean and obese mice. Interestingly the correlations were not restricted to the corresponding cellular compartment but showed systemic links. For example, 3-hydroxybutyrate in serum negatively correlated with BAL CD4^+^ T cells in lean mice, whereas urinary 3-hydroxybutyrate positively correlated with BAL CD4^+^ T cells in obese mice (Table S1). A relatively similar pattern of correlations was detected for BAL Tregs (CD4^+^CD25^+^Foxp3^+^), but 3-hydroxybutyrate in the lungs of obese mice positively correlated with BAL Tregs (Figure 4, Table 3). Further, 3-hydroxybutrate in several compartments correlated with various mLN and BAL CD8^+^ T cell populations in lean and obese mice (Table 4 and Table S1). Other metabolites, such as choline, taurine and creatine from a variety of tissues/biofluids were found to be significantly associated with several T cell populations in the lung airways and mLN. A detailed list of correlative metabolic markers is provided in Tables 3, 4 and S1. Significant correlations among metabolites and T cell populations may result from co-variation without indication of a mechanistic link, but it is also possible that detected correlations reveal underlying mechanisms directly or indirectly affecting T cell distribution and function in lean and obese mice.

**Table 3:** Lung metabolite correlation patterns with BAL T cell populations^a^.

BAL Cells	Lean	Obese
Total BAL cell number	creatine, glycerol, taurine	
CD4^+^ T cells	creatine, glycerol, phosphocholine, taurine	
CD4^+^CD25^+^ T cells	3-hydroxybutyrate, acetate, alanine,creatine, glycerol, lactate, phosphocholine, taurine	
CD4^+^CD25^+^FoxP3^−^ T cells	3-hydroxybutyrate, creatine, glycerol,lactate, phosphocholine, taurine	
CD4^+^CD25^+^Foxp3^+^ T cells	3-hydroxybutyrate, acetate, choline,creatine, glycerol, lactate, phosphocholine, taurine	3-hydroxybutyrate
CD4^+^FoxP3^+^ T cells	3-hydroxybutyrate, acetate, choline,creatine, glycerol, lactate, phosphocholine, taurine	3-hydroxybutyrate
CD4^+^FoxP3^hi^ T cells	3-hydroxybutyrate, acetate, choline,creatine, glycerol, lactate, phosphocholine, taurine	
CD8^+^CD25^+^ T cells	3-hydroxybutyrate, creatine	
CD8^+^D_b_NP_366-74_^+^ T cells		choline, glycerol
CD8^+^CD25^+^D_b_NP_366-74_^+^ T cells		acetate, alanine, choline, creatine, glucose, glycerol, lactate, leucine, methionine, phosphocholine, taurine, valine

a Underlined metabolites represent a significant negative correlation, and text without an underline indicates a significant positive association. Correlation analysis is based on a Pearson correlation matrix validated by 10,000 permutations. n = 8–9.

**Table 4:** Lung metabolite correlation patterns with mLN T cell populations^a^.

mLN Cells	Lean	Obese
Total mLN cell number		glucose
CD4^+^ T cells		glucose, glycerol
CD4^+^CD25^+^Foxp3^+^ T cells	alanine, leucine,valine	glucose
CD4^+^Foxp3^+^ T cells	leucine, valine	
CD4^+^Foxp3^hi^ T cells		glucose
CD8^+^ T cells		3-hydroxybutyrate, glucose, glycerol
CD8^+^D_b_NP_366-74_^+^ T cells		3-hydroxybutyrate, acetate, alanine, choline, creatine, glucose, glycerol, lactate, leucine, methionine, phosphocholine, valine
CD8^+^CD25^+^D_b_NP_366-74_^+^ T cells		3-hydroxybutyrate, acetate, alanine, choline, creatine, glucose, glycerol, lactate, leucine, methionine, taurine, valine phosphocholine

a Underlined text represents a significant negative correlation, and text without an underline indicates a significant positive association. Correlation analysis is based on a Pearson correlation matrix validated by 10,000 permutations. n = 8–9.

## Conclusion

Globally, 500 million adults are clinically obese, and the number of pathophysiological complications and identified health risks of excess adiposity continue to mount [2]. Although a number of innate and adaptive immune defenses are altered by obesity during influenza infection, explanatory mechanisms remain relatively undefined. Through metabolic assessment of urine and feces, we demonstrated that metabolic profiling can successfully distinguish uninfected lean and obese mice, naïve and infected mice, and lean and obese mice infected with influenza. Metabolic analysis of serum, WAT and livers also revealed differences in a variety of metabolic pathways in obese mice during the peak of influenza immune responses. Lastly, statistical relationships between T cell responses and tissue/biofluid metabolites were uncovered allowing for a thorough differential immune-metabolic characterization.

Metabolic profiling of urine and feces revealed greater levels of urinary taurine, ureidopropanoate, 1-methylnicotinamide, glucose and fecal choline in uninfected obese mice. Metabolic changes detected in the urine and feces may reflect local changes in the kidneys or gastrointestinal system, as well as systemic alterations in metabolism. To our knowledge, there are currently no reports defining how influenza infection alters metabolite profiles in the urine and feces of mice. During infection, acetylcarnitine, ascorbate, glucose and 3-hydroxybutyrate were elevated in the urine of obese mice. Of interest, a variety of lipid metabolites and propionate (a short chain fatty acid) [46] were detected at greater concentrations in the feces of obese mice at 2 dpi. Because these changes were not observed prior to infection, the increased levels of these metabolites are specific to the infection in obese mice (and not simply due to diet or obesity status). This finding may yield new hypotheses related to obesity and influenza infection, such as: Are there local changes in the gastrointestinal system of obese mice during influenza that may impact infection outcome? Does obesity induce alterations in the gut microbiota during influenza, potentially impacting infection responses (propionate can be produced by gut microbiota) [46]? Further, acetylcarnitine was detected at higher levels in the urine of obese mice at 6 dpi. Acetylcarnitine is the acetylated form of carnitine, which is utilized in fatty acid transport into mitochondria for subsequent β-oxidation. Therefore, various metabolites related to lipid metabolism were significantly altered in the feces and urine of obese mice, perhaps suggesting alterations in lipid metabolism contributes to differential infection responses in obese mice. Of interest, it has been demonstrated metabolic profiling of mouse serum revealed influenza infection alone causes changes in a number of lipid metabolites [30].

Ascorbate (vitamin C) was significantly higher in the urine of obese mice at 2 dpi. Excess levels of ascorbate in mice are controlled, in part, through urinary excretion [47], [48]. Unlike humans, mice have the ability to synthesize endogenous ascorbate [49], [50]. Further, during times of stress, mammals upregulate ascorbate biosynthesis [51]. Ascorbate fulfills a variety of physiological functions, including regulation of oxidative stress [50], [52]. Elevated ascorbate in the urine may be indicative of greater levels of oxidative stress in infected obese mice. Oxidative stress and vitamin C deficiency in mice can increase influenza infection pathology and mortality [50], [53], [54]. Further, influenza infection and obesity are independently associated with greater levels of oxidative stress [55], [56]. Therefore, it is likely that obesity exacerbates oxidative stress conditions during influenza infections, ultimately affecting infection outcome. Further, if vitamin C requirements are increased during infection in the obese, perhaps obese humans require greater ascorbate intake during influenza infection and should supplement during influenza seasons. It is interesting that ascorbate was detected at greater levels in the urine of obese mice, but it is unclear why elevated levels weren’t detected in the serum or the liver (primary site of synthesis). Perhaps, at 9 dpi, when serum and livers were harvested, ascorbate in obese mice returned to similar levels as in lean mice.

Another interesting finding revealed by metabolic profiling was that glucose was significantly elevated in the urine of obese mice prior to infection, and at 2 and 6 dpi. Glucose intolerance often results in glucosuria [42], [57]. Further, obese mice had greater levels of glucose in the serum and liver at 9 dpi compared with lean mice. It has been extensively demonstrated that diet-induced and genetic obese mice exhibit elevated blood glucose levels [58], [59]. However, tissue and circulating glucose levels in obese mice have never been measured during the context of an influenza infection. Chronically elevated glucose can have a variety of pathological effects, including glycation products, oxidants, hyperosmolarity and perturbations in cell signaling pathways [59]. Therefore, it would be interesting to investigate influenza severity in a model in which obese mice have normalized insulin sensitivity and glucose tolerance [28].

In recent years, an abundance of research has focused on how metabolism is a critical regulator of immune cell function [60]. Manipulation of nutrient availability in culture or genetic manipulation of genes that regulate metabolic pathways can have a profound impact on immune activity and disease outcome [4], [60]. However, it is relatively unclear how metabolites and nutrients in the immune cell microenvironment may alter cellular metabolism, distribution and function during the context of an infection. Therefore, we took advantage of this comprehensive metabolic analysis and assessed significant interactions between T cell populations and metabolites. A number of immune-metabolic correlations were uncovered. For example, lung choline and phosphocholine were positively associated with BAL Tregs and other CD4^+^ T cell populations in lean mice but not in obese mice. Perhaps, perturbations in choline/phosphocholine metabolism may impact T cell responses in obese mice. These findings could be indicative of direct or indirect T cell-metabolite interactions, although it is also possible that these associations are biologically insignificant. Nonetheless, identification of BAL and mLN T cell-metabolite correlates provides a more dynamic and global assessment of the consequences of obesity during influenza infection.

The urinary and fecal data are particularly interesting given the non-invasive nature of these bio-samples and the high degree of separation detected between naïve and infected, lean and obese mice. Numerous reports have established metabolic profiling of urine can identify biomarkers and metabolic matrices that can distinguish disease states, with the capability of ultimately guiding treatment [61]–[63]. At 2 dpi, prior to significant weight loss and any obvious signs of sickness, we identified a unique metabolic fingerprint in the urine and feces of influenza infected obese mice, consisting of perturbations in lipid, nucleotide, carbohydrate, microbial, and vitamin metabolism. Given the rapidity of influenza transmission during epidemics and pandemics and the sudden onset of severe symptoms in infected individuals [14], identification of a metabolic profile unique to an early infection time point holds widespread implications for personal and public health. We have identified a metabolic signature that could be used to predict influenza infection status even prior to any obvious signs of illness. Further testing can determine if this signature is unique to influenza infection rather than a generalized response to an infection or inflammation.

Taken together, this investigation establishes metabolic profiling as a useful tool for characterizing infection responses during influenza and identifying potential pathways and mechanisms contributing to altered immunity in obese mice. Teasing apart differential responses during influenza infection is key to understanding the mechanisms driving greater disease severity in obese mice compared with lean mice. Further utilization of metabolic profiling as a complimentary tool to immunological measures of infection outcome could help advance the current knowledge of the response to influenza infection in other rodent research models and may have potential applications in clinical and research settings.

## Supporting Information

Table S1 Correlation patterns between ^1^H NMR data and BAL or mLN T cell populations.
(DOCX)
