# Supplementary material for: 1H NMR-Based Profiling Reveals Differential Immune-Metabolic Networks during Influenza Virus Infection in Obese Mice
Source: PLoS One. 2014 May 20;9(5):e97238. doi: 10.1371/journal.pone.0097238 (PMC4028207; doi:10.1371/journal.pone.0097238)
Supplement: Table S1 — Correlation patterns between 1H NMR data and BAL or mLN T cell populations. (DOCX) [file pone.0097238.s001.docx]

**Table S1.** Correlation patterns between ^1^H NMR data and BAL or mLN T cell populations^a^

| *Liver NMR* |  | Lean | Obese |
| --- | --- | --- | --- |
| *BAL cells* | **Total BAL cell number** | **glucose, leucine** |  |
|  | **CD4^+^ T cells** | **acetate, alanine, glucose, lactate, leucine** | **acetate, glucose, glutamine, isoleucine** |
|  | **CD4^+^CD25^+^ T cells** | **acetate, alanine, glucose, glycerol, lactate, leucine** |  |
|  | **CD4^+^CD25^+^FoxP3^-^ T cells** | **acetate, alanine, choline, glucose, glycerol, lactate, leucine** |  |
|  | **CD4^+^FoxP3^+^ T cells** | **glucose** |  |
|  | **CD4^+^FoxP3^hi^ T cells** | **glucose** |  |
|  | **CD4^+^CD25^+^Foxp3^+^ T cells** | **glucose** |  |
|  | **CD8^+^CD25^+^ T cells** | **glucose** | **acetate, choline, glucose, glutamine, isoleucine, lactate** |
|  | **CD8^+^CD25^+^D_b_NP_366-74_^+^ T cells** |  | **alanine, aspartate, choline, glucose, glutamate, glycerol** |
| *mLN cells* | **CD4^+^ T cells** | **choline** |  |
|  | **CD4^+^CD25^+^ T cells** | **acetate, choline** |  |
|  | **CD4^+^CD25^+^Foxp3^+^ T cells** | **acetate, choline** |  |
|  | **CD4^+^Foxp3^+^ T cells** | **choline** |  |
|  | **CD4^+^Foxp3^hi^ T cells** | **choline** |  |
|  | **CD4^+^CD25^+^Foxp3^-^ T cells** | **acetate, choline** |  |
|  | **CD3^+^CD8^+^ T cells** | **choline** |  |
|  | **CD8^+^D_b_NP_366-74_^+^ T cells** |  | **choline, glucose** |
|  | **CD8^+^CD25^+^D_b_NP_366-74_^+^ T cells** |  | **choline, glucose, glutamate** |
| *WAT NMR* |  | **Lean** | **Obese** |
| *BAL cells* | **CD4^+^ T cells** |  | **choline, lactate, phosphocholine, taurine** |
|  | **CD4^+^Foxp3^+^ T cells** |  | **phosphocholine** |
|  | **CD4^+^FoxP3^hi^ T cells** |  | **choline, phosphocholine, taurine** |
|  | **CD8^+^CD25^+^ T cells** |  | **alanine, glycerol, leucine** |
| *mLN cells* | **Total mLN cells** | **taurine** |  |
|  | **CD3^+^CD4^+^ T cells** | **glycerol, lactate, taurine** |  |
|  | **CD4^+^CD25^+^Foxp3^-^ T cells** |  | **leucine** |
|  | **CD3^+^CD8^+^ T cells** | **glycerol, lactate, taurine** |  |
| *Feces NMR* |  | **Lean** | **Obese** |
| *BAL cells* | **Total BAL cell number** |  | **deoxycholic acid** |
|  | **CD4^+^CD25^+^ T cells** |  | **deoxycholic acid** |
|  | **CD4^+^CD25^+^FoxP3^-^ T cells** |  | **deoxycholic acid** |
|  | **CD4^+^CD25^+^Foxp3^+^ T cells** |  | **deoxycholic acid** |
|  | **CD4^+^FoxP3^+^ T cells** |  | **deoxycholic acid** |
|  | **CD4^+^FoxP3^hi^ T cells** |  | **deoxycholic acid** |
|  | **CD8^+^CD25^+^ T cells** |  | **deoxycholic acid** |
|  | **CD8^+^CD25^+^D_b_NP_366-74_^+^ T cells** | **acetate** |  |
|  | **CD8^+^D_b_NP_366-74_^+^ T cells** | **acetate** |  |
| *mLN cells* | **CD3^+^CD4^+^ T cells** | **deoxycholic acid** |  |
|  | **CD4^+^CD25^+^Foxp3^-^ T cells** |  | **deoxycholic acid** |
|  | **CD3^+^CD8^+^ T cells** | **deoxycholic acid** | **deoxycholic acid** |
| *BALF NMR* |  | **Lean** | **Obese** |
| *mLN cells* | **CD8^+^D_b_NP_366-74_^+^ T cells** | **lactate** |  |
|  | **CD8^+^CD25^+^D_b_NP_366-74_^+^ T cells** | **lactate** |  |
| *Serum NMR* |  | **Lean** | **Obese** |
| *BAL cells* | **CD3^+^CD4^+^ T cells** | **3-hydroxybutyrate, acetone** |  |
|  | **CD4^+^CD25^+^ T cells** | **3-hydroxybutyrate, acetone** |  |
|  | **CD4^+^CD25^+^Foxp3^+^ T cells** |  | **3-hydroxybutyrate** |
|  | **CD4^+^CD25^+^FoxP3^-^ T cells** | **3-hydroxybutyrate, acetone** |  |
|  | **CD4^+^FoxP3^+^ T cells** |  | **3-hydroxybutyrate** |
|  | **CD4^+^FoxP3^hi^ T cells** |  | **3-hydroxybutyrate** |
|  | **CD3^+^CD8^+^ T cells** | **3-hydroxybutyrate, acetone** |  |
|  | **CD8^+^CD25^+^ T cells** | **3-hydroxybutyrate, acetone** |  |
| *mLN cells* | **CD4^+^CD25^+^Foxp3^-^ T cells** | **acetone** |  |
| *Urine NMR* |  | **Lean** | **Obese** |
| *BAL cells* | **Total BALF cell number** | ***N*-acetyl glycoprotein** | **glucose** |
|  | **CD3^+^CD4^+^ T cells** | ***N*-methylnicotinamide** | **3-hydroxybutyrate, glucose, *N*-acetyl glycoprotein, taurine** |
|  | **CD4^+^CD25^+^ T cells** | ***N*-methylnicotinamide, trimethylamine** | **2-oxovalerate, ascorbate, creatine, creatinine, glucose** |
|  | **CD4^+^CD25^+^FoxP3^-^ T cells** | **guanidinoacetate, *N*-methylnicotinamide, trimethylamine** | **2-oxovalerate, ascorbate, creatine, creatinine** |
|  | **CD4^+^CD25^+^Foxp3^+^ T cells** |  | **2-oxovalerate, ascorbate, creatinine** |
|  | **CD4^+^FoxP3^+^ T cells** |  | **glucose** |
|  | **CD4^+^FoxP3^hi^ T cells** |  | **2-oxovalerate** |
|  | **CD3^+^CD8^+^ T cells** | **3-hydroxybutyrate** | **glucose** |
|  | **CD8^+^CD25^+^ T cells** | **3-hydroxybutyrate** |  |
|  | **CD8^+^D_b_NP_366-74_^+^ T cells** | **creatine, creatinine, indoxylsulfate, *N*-acetyl glycoprotein, phenoacetylglycine, taurine** | **glucose** |
|  | **CD8^+^CD25^+^D_b_NP_366-74_^+^ T cells** | **ascorbate, creatine, creatinine, indoxylsulfate, *N*-acetyl glycoprotein, phenoacetylglycine, taurine** | **glucose, guanidinoacetate, indoxylsulfate, *p* cresol glucuronide, phenoacetylglycine** |
| *mLN cells* | **mLN cell number** |  | **2-oxovalerate, creatinine** |
|  | **CD3^+^CD4^+^ T cells** | ***N*-methylnicotinamide, trimethylamine** | **2-oxovalerate, creatinine, glucose** |
|  | **CD4^+^CD25^+^ T cells** |  | **2-oxovalerate, ascorbate, creatinine, dimethylamine, glucose, *N*-methylnicotinamide** |
|  | **CD4^+^CD25^+^Foxp3^-^ T cells** | **indoxylsulfate** | **2-oxovalerate, ascorbate, creatinine** |
|  | **CD4^+^CD25^+^Foxp3^+^ T cells** |  | **2-oxovalerate ascorbate, creatinine, dimethylamine, glucose, *N*-methylnicotinamide** |
|  | **CD4^+^Foxp3^+^ T cells** |  | **2-oxovalerate, ascorbate, creatinine, dimethylamine, glucose, *N*-methylnicotinamide** |
|  | **CD4^+^Foxp3^hi^ T cells** | **indoxylsulfate** | **Ascorbate, creatinine, dimethylamine, glucose, *N*-methylnicotinamide** |
|  | **CD3^+^CD8^+^ T cells** | ***N*-methylnicotinamide, trimethylamine** | **2-oxovalerate, creatinine, glucose, phenoacetylglycine** |
|  | **CD8^+^D_b_NP_366-74_^+^ T cells** | **3-hydroxybutyrate** | **guanidinoacetate, indoxylsulfate, *p* cresol glucuronide, phenoacetylglycine** |
|  | **CD8^+^CD25^+^D_b_NP_366-74_^+^ T cells** |  | **guanidinoacetate, indoxylsulfate, *p* cresol glucuronide, phenoacetylglycine** |

^a^Underlined text represents a significant negative correlation, and text without an underline indicates a significant positive association. All ^1^H NMR data are from tissues harvested at 9 dpi or urine and feces harvested at 6 dpi. Correlation analysis is based on a Pearson correlation matrix validated by 10,000 permutations. n=8-9.
